# 2D and 3D similarity landscape analysis identifies PARP as a novel off-target for the drug Vatalanib

**DOI:** 10.1186/s12859-015-0730-x

**Published:** 2015-09-24

**Authors:** Bjoern-Oliver Gohlke, Tim Overkamp, Anja Richter, Antje Richter, Peter T. Daniel, Bernd Gillissen, Robert Preissner

**Affiliations:** 1 Structural Bioinformatics Group, Charite - University Medicine Berlin & ECRC, Lindenberger Weg 80, 13125 Berlin, Germany; 2 Department of Hematology, Oncology and Tumor Immunology, University Medical Center Charité, Campus Berlin-Buch, Humboldt University Berlin, Berlin, Germany; 3 German Cancer Consortium (DKTK) and German Cancer Research Center (DKFZ), Heidelberg, Germany; 4 Clinical and Molecular Oncology, Max Delbrück Center for Molecular Medicine, 13125 Berlin-Buch, Berlin, Germany

**Keywords:** Bioinformatics, Drug action, Drug discovery, Polyadenylation, Vascular endothelial growth factor (VEGF), 3D similarity landscapes, Drug-discovery, Vatalanib, PARP

## Abstract

**Background:**

Searching for two-dimensional (2D) structural similarities is a useful tool to identify new active compounds in drug-discovery programs. However, as 2D similarity measures neglect important structural and functional features, similarity by 2D might be underestimated. In the present study, we used combined 2D and three-dimensional (3D) similarity comparisons to reveal possible new functions and/or side-effects of known bioactive compounds.

**Results:**

We utilised more than 10,000 compounds from the SuperTarget database with known inhibition values for twelve different anti-cancer targets. We performed all-against-all comparisons resulting in 2D similarity landscapes. Among the regions with low 2D similarity scores are inhibitors of vascular endothelial growth factor receptor (VEGFR) and inhibitors of poly ADP-ribose polymerase (PARP). To demonstrate that 3D landscape comparison can identify similarities, which are untraceable in 2D similarity comparisons, we analysed this region in more detail. This 3D analysis showed the unexpected structural similarity between inhibitors of VEGFR and inhibitors of PARP. Among the VEGFR inhibitors that show similarities to PARP inhibitors was Vatalanib, an oral “multi-targeted” small molecule protein kinase inhibitor being studied in phase-III clinical trials in cancer therapy. An *in silico* docking simulation and an *in vitro* HT universal colorimetric PARP assay confirmed that the VEGFR inhibitor Vatalanib exhibits off-target activity as a PARP inhibitor, broadening its mode of action.

**Conclusion:**

In contrast to the 2D-similarity search, the 3D-similarity landscape comparison identifies new functions and side effects of the known VEGFR inhibitor Vatalanib.

**Electronic supplementary material:**

The online version of this article (doi:10.1186/s12859-015-0730-x) contains supplementary material, which is available to authorized users.

## Background

Drugs often not only interact with their intended target but also with so-called off-targets, thereby causing side-effects [1]. Prediction of side-effects is still a big challenge during drug design and studies have shown the potential of computational methods for target and off-target analysis [2, 3]. These studies deal with pathway- and network-based approaches combined with the chemical structure of small molecule compounds regarding their binding site at the target protein [4–6].

To identify compound similarities it is important to take a detailed look at 2D- and 3D-similarities [7]. 2D structural similarity algorithms were generated to predict and create a drug-target adverse drug reactions (ADR) network [3, 8]. These 2D-fingerprints represent the structure and properties of small molecules by a bit or integer string. Although several methods exist to measure the similarity between 2D fingerprints, the Tanimoto coefficient has been proven to be reliable [9, 10]. However, several problems can occur while working with fingerprints: size of compounds as well as functional groups or side chains have an impact on the similarity calculations. Hence, functional and structural features of compounds can be neglected. These problems can be overcome by using 3D similarity search methods.

Non-commercial drug- or target-related databases, which have been established in the last decade, can be used for 2D and 3D comparisons. Millions of compounds can be found in databases like ChEMBL [11] or PubChem [12] and their availability can be verified via the ZINC database [13].

We recently established our SuperTarget database, which was developed with the intention to accentuate drug–target interactions and to provide references to other resources for more elaborate analysis [14]. The SuperTarget database contains a core dataset of about 330,000 drug-target interactions, of which about 310,000 interactions have calculated binding affinity data [15] and were used to compare 2D and 3D structures of promising anticancer drugs.

Among these drugs are inhibitors of the poly ADP-ribose polymerase (PARP). PARP binds to single-strand DNA breaks and plays a critical role in cell recovery from DNA damage. PARP inhibitors show activities not only in cancer therapy but are also being evaluated for the treatment of stroke, myocardial infarction and other diseases. Additional promising anticancer drugs, which can be found in the SuperTarget database, are inhibitors of the vascular endothelial growth factor receptor (VEGFR). The approved VEGFR inhibitor Vatalanib (PTK787 or PTK/ZK) is currently studied in several phases of clinical trials for different cancer therapies [16–18]. Vatalanib is an oral “multi-targeted” small molecule protein kinase inhibitor that binds to the intracellular kinase domain of all VEGF receptor subtypes, thereby inhibiting angiogenesis [19]. In addition, it binds to c-KIT and platelet-derived growth factor receptor (PDGFR) but with lower affinity.

While applying a 3D similarity landscape analysis on inhibitors for different cancer targets by using the SuperTarget database, we found unexpected similarities between PARP and VEGFR inhibitors, which could not be detected by 2D similarity searches. As a proof of concept of our similarity landscape analysis, both *in silico* and *in vitro* assays confirmed Vatalanib’s off-target activity as a PARP inhibitor. In this paper we provide a combined approach of 2D and 3D similarity landscapes for target and off-target analysis, which can be applied to a larger number of targeted anti-cancer therapeutics.

## Methods

*2D-similarity* - 2D-similarity was calculated with the Tanimoto coefficient for pairs of compounds, i.e. inhibitors [20]. For many of these inhibitors the half-maximal inhibitory concentration (IC_50_), effective concentration (EC_50_), or dissociation constant (Kd) values are listed in the SuperTarget database. These known affinities of the inhibitors were the basis of our filter algorithm and, to avoid unwanted and therefore incomputable off-target effects, only interactions described by binding affinities <10 μM (IC_50_, EC_50_ or Kd) were considered for further analysis. Using this filter method, we identified nearly 10,000 inhibitors for the twelve different anti-cancer targets. To compare these inhibitors, a combination of fingerprint 2 (FP2; http://openbabel.org/wiki/FP2) and fingerprint 4 (FP4) were calculated. FP2 is used to compare small molecules; it links linear segments of a fragment up to seven atoms to an index and considers atoms and bonds of a fragment and whether a complete ring exists. Based on these calculations, fragments are assigned to set bits in a 1,024 bit vector. FP4 uses SMART patterns of functional groups of the small molecules to set bits in a bit vector. The calculated fingerprints were subsequently compared by the Tanimoto similarity measure for bit strings [7]. The Tanimoto coefficient is based on a similarity ratio and can assume values between zero and one, indicating no similarity or identical structures respectively. It is calculated using the bits of the binary fingerprint vectors set to one in molecule A and molecule B:

$$ \mathrm{Tanimoto}\ {\mathrm{coefficient}}_{A,B}=\frac{AB}{A+B - AB} $$

where AB is the number of bits set to one in both molecules, A is the number of bits set to one in molecule A and B is the number of bits set to one in molecule B.

Another method to calculate the Tanimoto coefficient are the extended-connectivity fingerprints (ECFP) [21]. These are used to cover the calculated 2D-similarity by OpenBabel fingerprints, which belong to the class of radial fingerprints and are based on the Morgan algorithm [22]. To calculate the extended-connectivity fingerprints the cheminformatics toolkit of ChemAxon was used (JChem compr (14.10.20.0), 201n (2014), ChemAxon (http://www.chemaxon.com)).

*3D-similarity* - For 3D-similarity comparisons pre-calculated conformers are superimposed using the Kabsch algorithm [23]. Based on the normalised set of atoms in a coordinate system the centres of mass for both conformers were calculated and superimposed. Then the principal axes of inertia are estimated and aligned. Thereby the possible rotations are strongly reduced and only four orientations have to be considered. For every orientation a mapping of atom pairs was performed whereupon atoms were fitted to each other with the smallest possible distance. Because for atom pair assignment a maximal distance threshold is applied, not every atom is assigned. The rotation with the highest amount of mapped pairs was used for further calculations. The normalised variant with the most minimal distance is chosen if more than one rotation with the same amount of mapped atom pairs exists. For this mapping a root-mean-square-deviation (rmsd) was calculated and further optimised.

$$ \mathrm{rmsd}(M)=\sqrt{\frac{1}{k}{\displaystyle \sum_{i=1}^k} dist{\left({a}_i^M,{b}_i^M\right)}^2} $$

Every molecule’s conformation was compared with each conformation of the second molecule, resulting in up to 2,500 separately calculated rmsd values. Here, only the smallest rmsd value, i.e. the best superposition of the compounds, was stored.

*Ligand Docking* - The docking study was performed by using LibDock, a high-throughput docking algorithm for library design and library prioritisation. This docking program was provided by Accelrys Discovery Studio (http://accelrys.com). The algorithm positioned ligands in the protein’s active site based on polar and non-polar interaction sites.

*MCF-7 cell lines* - Breast cancer cell lines MCF-7 were cultured in RPMI-1640 medium supplemented with 10 % inactivated FBS, 100 U/ml penicillin and 0.1 mg/ml streptomycin. Cells were cultured at 37 °C with 5 % CO_2_ in a fully humidified atmosphere.

*IC*_*50*_ *values of PARP inhibitors* - For the determination of *IC*_*50*_ values of Vatalanib and Compound 1 we used the HT universal colorimetric PARP assay kit with histone-coated strip wells (Trevigen, USA). Absorbance was measured in a Sunrise microplate reader (Tecan, Switzerland) at 450 nm.

*γH2AX foci analysis* - For immunofluorescence microscopic analyses, MCF-7 cells were grown on coverslips. 24 h post treatment with 0 (control), 1, 10, and 100 μM Compound 1 or Vatalanib, cells were washed in PBS, fixed in 3 % paraformaldehyde/PBS (15 min), permeabilised with 0.5 % Triton-X 100/PBS (2 min) and blocked in 5 % fetal bovine serum for 60 min at room temperature. After incubation with anti-phospho-Histone H2A.X (Ser139) clone JBW301 (mouse monoclonal IgG from Millipore, Billerica, MA, USA) overnight at 4 °C, cells were incubated with Alexa Fluor 488-labelled chicken anti-mouse IgG secondary antibody (Molecular Probes, The Netherlands) for 2 h at room temperature and counterstained with DAPI. Images of γH2AX foci and DAPI-labelled nuclei were acquired with a fluorescent microscope (BX50; Olympus, Germany) equipped with a 40×/0.75 objective lens (UPlanFL; Olympus, Germany) and a camera (micropublisher 5.0 RTV; QImaging, Canada) with Openlab software (Perkin Elmer, Germany).

## Results

**Fig. 1:**
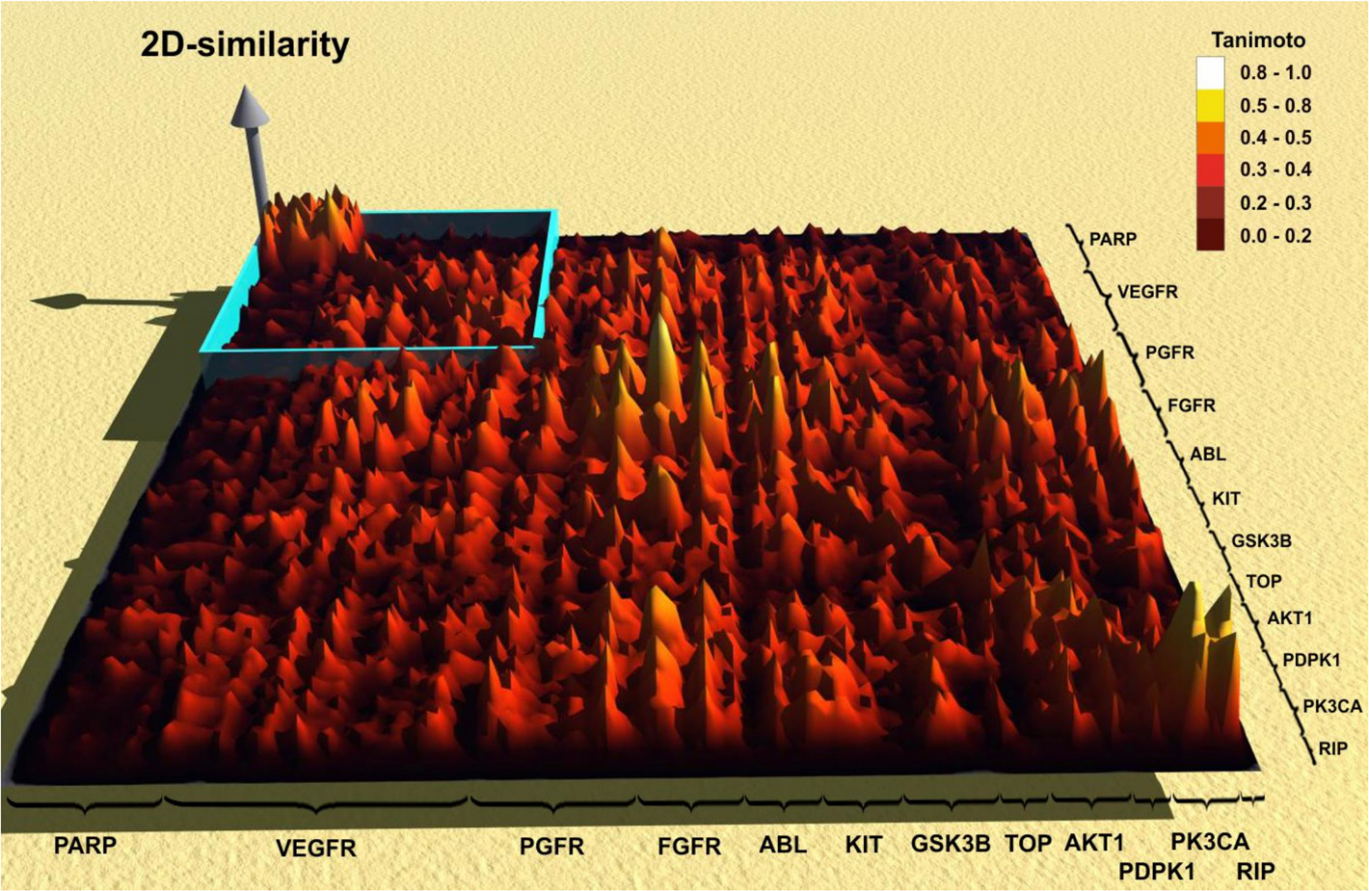
2D similarity landscape. 2D similarity landscape of about 10,000 inhibitors for the twelve anti-cancer targets. High similarity, calculated by the Tanimoto score, is visualised by mountains (white-yellow) and low similarity by valleys (dark red). We focused on the similarity between PARP and VEGFR inhibitors (blue square, upper left corner

First, we performed an *in silico* screening for a variety of known inhibitors against twelve promising anti-cancer targets (listed in Fig. 1) using our SuperTarget database (http://bioinformatics.charite.de/supertarget) [15]. The overall 2D-similarity of about 10,000 inhibitors was then displayed in heat-maps (Additional file 1: Figure S1), where the values are coloured according to the similarity of the analysed inhibitors as calculated by the Tanimoto score: high similarity is displayed in red and low similarity in yellow data points. To allow for a better access to these values, we represent these heat-maps as landscapes of similarity. Here, high similarity is represented by mountains and low similarity by valleys (see Fig. 1). Within each group and in between groups, inhibitors display similarity as indicated by high Tanimoto scores depicted by ridges and mountains. In contrast, the 2,547 VEGFR inhibitors showed only little structural similarity to the other classes of inhibitors, but especially to the 1,080 PARP inhibitors (Fig. 1, upper left corner).

Because 2D-similarity analyses often neglect important structural and functional features, we expanded our comparison to 3D-superpositions measured by the root-mean-square deviation (rmsd) based on the Kabsch algorithm. Although current computers calculate 3D-comparisons of compounds relatively fast, it would still take months to compare all inhibitors with each other. We therefore focused on the 3D-structural comparison of VEGFR and PARP inhibitors, which showed only little structural similarity in the 2D-similarity analyses. For this comparison up to 50 conformers were calculated by using Accelrys Discovery studio 3.5 (Accelrys Software Inc., Discovery Studio Modeling Environment, Release 3.5, San Diego: Accelrys Software Inc., 2012). To create diverse ligand conformations, the ‘fast’ search method was used to generate multiple low-energy conformations. The rmsd calculations are based on overlaying the anchor points of both conformers and to rotate at single bonds (degrees of freedom) to minimise the rmsd. We arbitrarily chose a 5 % quantile (rmsd of 0.215) to evaluate the similarity of related 3D structures and analysed only compounds with both low rmsd (high similarity) and low Tanimoto scores (low 2D-similarity).

**Fig. 2:**
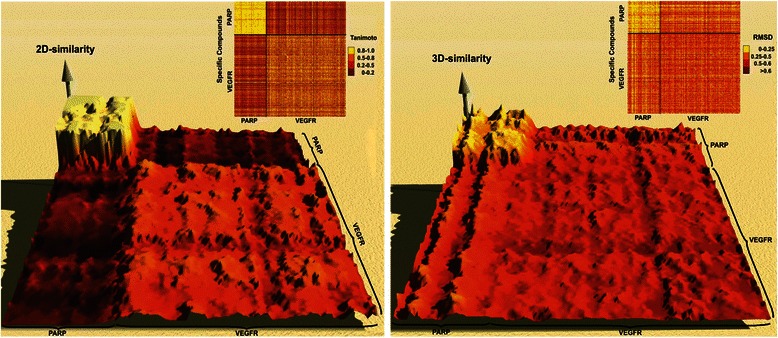
Similarity comparison of compounds of PARP to VEGFR. Comparison of PARP- (upper left corners) and VEGFR- (lower right corners) inhibitors displayed in heat-maps and landscapes. 2D-similarity (left) based on the Tanimoto coefficient, 3D-similarity (right) based on rmsd values. High similarity is visualised in yellow and low similarity in dark red. To display 3D similarity as mountains the rmsd values were transformed by the following formula: rmsd-score = overlapped atoms/possible overlapped atoms x e^-rmsd^. Points with small RMSD are depicted higher than ones with large RMSD. 3D-similarity landscape analysis reveals two ridges along the left and the upper side of the graph

The resulting overall 2D- and 3D-structural similarities of VEGFR and PARP inhibitors were again displayed in heat-maps and as landscapes of similarity (Fig. 2). The 2D-similarity clearly depicts that the inhibitory compounds of VEGFR and PARP are similar within each group, but that there is only little similarity detectable between both classes of inhibitors. The 3D-screening results are visualised in a similar heat-map and corresponding landscape that shows overall reduced similarity of inhibitors within each group. Nevertheless, selected PARP inhibitors display high similarity when compared to the VEGFR inhibitors. Interestingly, there is a remarkable similarity between some inhibitors of both classes (rmsd values between 0.1 and 0.3) depicted by the ridges, which cannot be seen in the 2D-similarity landscape, thereby proving the importance of additional 3D superposition analysis.

**Fig. 3:**
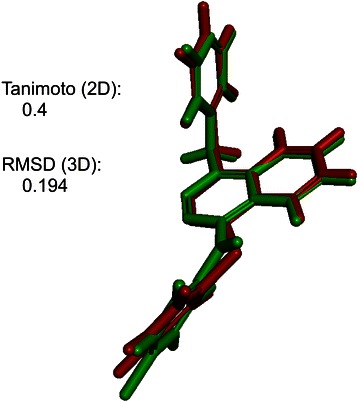
3D superposition of Vatalanib and Compound 1. 3D comparison of Vatalanib (green) and Compound 1 (red)

Among these similar inhibitors (for a more detailed list see Additional file 2: Table S1), we found Vatalanib (N-(4-chlorophenyl)-4-(pyridin-4-ylmethyl)phthalazin-1-amine; CID 151194) an approved inhibitor for VEGFR, which showed similarity to a well-known PARP inhibitor (1-benzyl-4-(1-oxidopyridin-1-ium-2-yl) sulfanylphthalazine; CID 6413221) [24], hereinafter referred to as Compound 1 (Fig. 3). Based on the fact that Vatalanib is a well-known drug, we decided to further investigate pair of compounds.

The 3D screening identifies Vatalanib as a potential inhibitor of PARP (rmsd: 0.194) whereas OpenBabel fingerprints calculated a low Tanimoto score of 0.4 and therefore failed to recognise the similarity of these two compounds. To analyse in more detail if other 2D fingerprints uncover the similarity between Vatalanib and Compound 1, Tanimoto scores were likewise calculated by extended connectivity fingerprints (ECFP), which result in a score of 0.32 for Compound 1 and Vatalanib (data matrix not shown). In addition, MACCS and FP3 fingerprints also computed low Tanimoto scores of 0.37 and 0.33, respectively. This confirmed that 2D analyses are unsuccessful in identifying the similarity between Vatalanib and Compound 1.

**Fig. 4:**
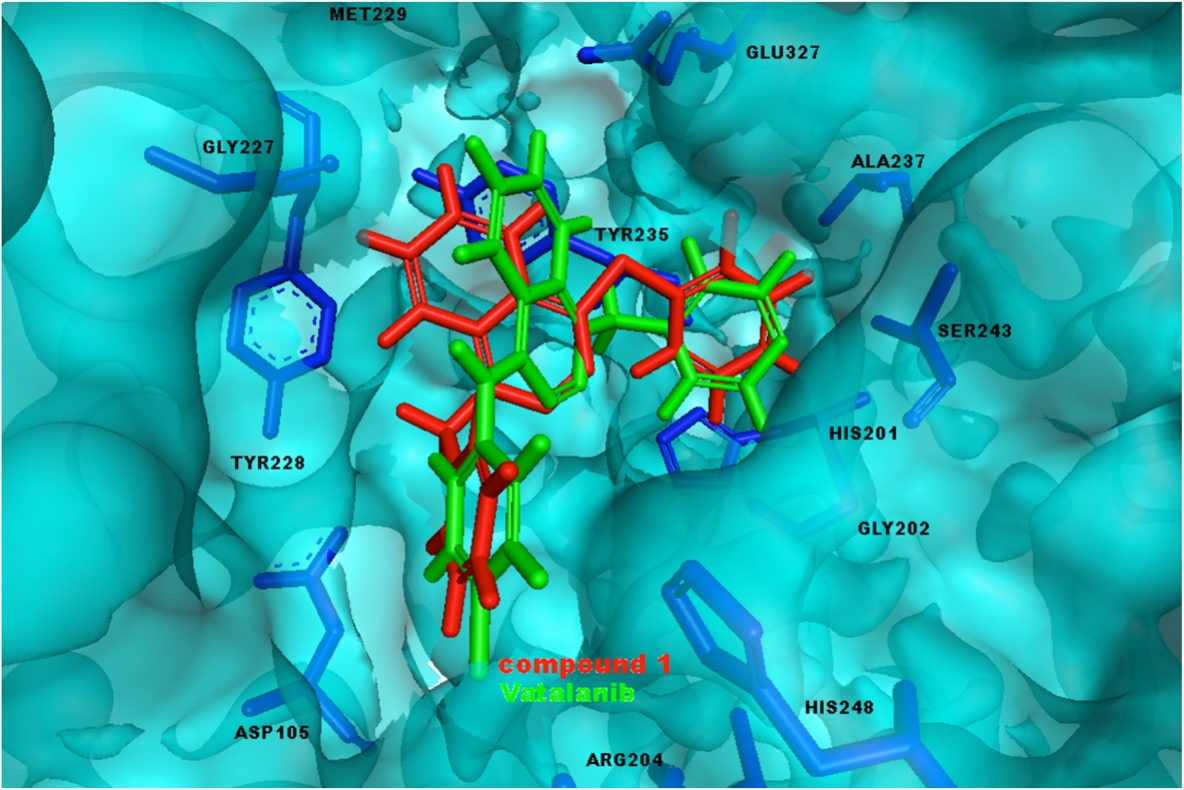
Best docking positions of Vatalanib and Compound 1 into PARP. Best docking positions of Vatalanib (green) and Compound 1 (red) into the active site of PARP

To verify Vatalanib’s function as an inhibitor of PARP, we first performed an *in silico* docking simulation to analyse the binding of both compounds into the active site of PARP (Fig. 4). The docking was performed by using the 3L3L PDB structure. PARP catalyses the NAD-dependent addition of poly (ADP-ribose) (PAR) onto various cytoplasmic and nuclear proteins, and PARP inhibitors are thought to compete with the enzyme substrate NAD+ at the active site. The high spatial similarity is displayed by overlapping both compounds as well as docking them to PARP. By using the high-throughput docking algorithm LibDock [25] the best docking positions of both compounds were calculated. In addition the docking score of 3-AB was calculated as a reference. According to the integrated scoring function, the best-ranked poses for Vatalanib and Compound 1 have comparable LibDock scores of 114.8 and 128.2, respectively, showing that Vatalanib, like Compound 1, fits neatly into the active site of PARP. Both structures have a higher docking score than the reference structure 3-AB with a docking score of 86.45 for the best-suited pose.

**Fig. 5:**
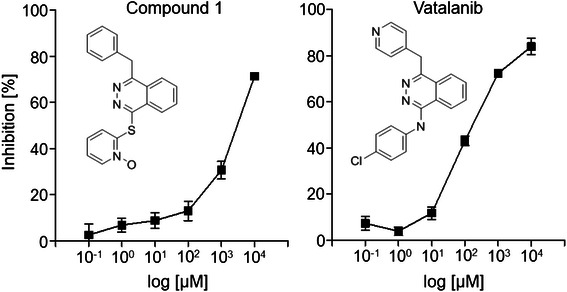
Colorimetric readout of the inhibition curves for Compound 1 and Vatalanib Graphical representation of the colorimetric readout of the inhibition curves for Compound 1 and Vatalanib. Percentage of inhibition was calculated as ([normal activity – inhibited activity] / normal activity) x 100 %. Each point represents the mean ± s.d. from triplicates

To confirm our computational hypothesis that Vatalanib also targets PARP, we next compared the IC_50_ values of both compounds. IC_50_ values were determined by the use of the HT universal colorimetric PARP assay kit, which measures the incorporation of biotinylated poly(ADP-ribose) onto histone proteins. Both compounds inhibited PARP activity in a concentration-dependent manner with IC_50_ values of about 3,000 μM and about 200 μM for Compound 1 and Vatalanib, respectively (Fig. 5).

To confirm inhibition of PARP and to rule out *in vivo* vs. *in vitro* activity discrepancies, i.e. differences of PARP expression in cells vs. an isolated PARP enzyme, we analysed accumulation of DNA damage in a human breast cancer cell line upon treatment with Compound 1 and Vatalanib. Because of PARP's involvement in DNA strand break repair, its inhibition has been proposed to lead to double-strand break (DSB) formation [26]. These DSBs induce phosphorylation of histone H2AX on Ser-139 at sites flanking the breakage [27, 28]. Therefore, we analysed whether treated cells accumulate phosphorylated H2AX, denoted as γH2AX, which provides a common marker for DNA damage in vitro [26, 29].

**Fig. 6:**
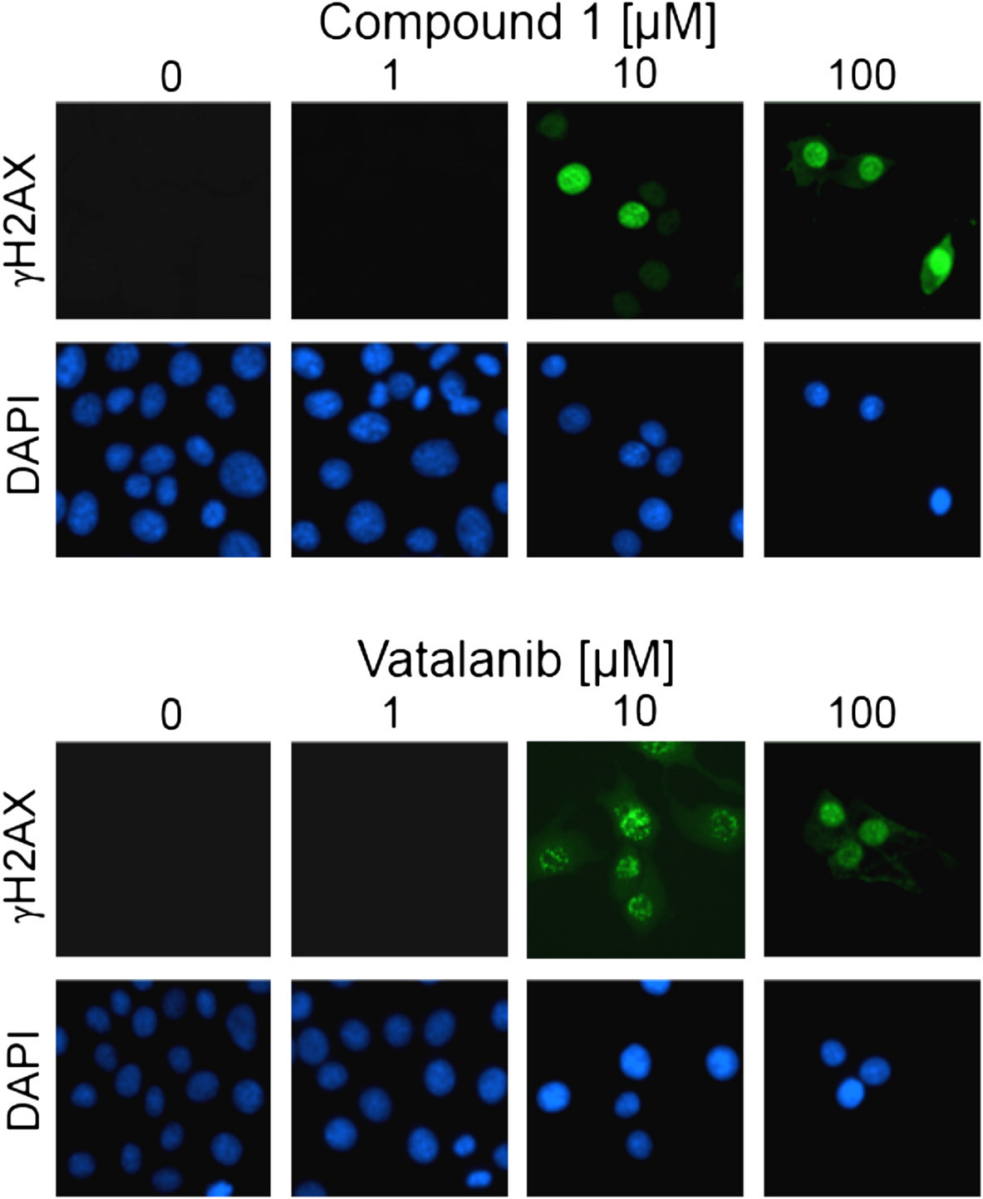
Immunofluorescence of MCF-7 cell lines. Immunofluorescence of MCF-7 cell lines 24 h after treatment with increasing concentrations of Compound 1 (top) and Vatalanib (bottom). Accumulation of γH2AX foci in green, 4’,6’-diamino-2-phenylindole (DAPI) staining in blue

MCF-7 cells were treated with increasing concentrations of Compound 1 and Vatalanib, incubated for 24 h and stained for γH2AX. Green immunofluorescence (Fig. 6) indicates accumulation of γH2AX foci after treatment with concentrations of 10 and 100 μM of Compound 1 or Vatalanib. Treatment with either 10 μM of Compound 1 or 10 μM of Vatalanib is sufficient to cause accumulation of γH2AX foci, indicating accumulation of DNA damage resulting from PARP inhibition.

## Discussion

2D fingerprints similarity search methods are widely used approaches in the discovery of novel molecules with high affinity to specific targets and, despite the fact that molecules are active in three dimensions, surprisingly powerful [30]. In this study we used the freely available software packages OpenBabel and ChemAxon to analyse the 2D-similarity of about 10,000 inhibitors against twelve promising anti-cancer targets. Among these VEGFR and PARP inhibitors showed only little structural similarity, however, similarity by 2D might be underestimated [31].

Accurate target prediction can be achieved by combining different measures of chemical similarity based on both chemical structure and molecular shape [32]. Furthermore it has been shown recently that the combination of a 2D similarity search and a 3D shape/flexibility-based similarity search led to an increased hit rate [33]. Therefore 3D-similarity of VEGFR and PARP inhibitors was then analysed by a proprietary 3D-superpostion algorithm, which produces reproducible results because of pre-calculated conformers for every compound. The 3D-similarity method in combination with 2D-similarity comparison performs quite well by applying a 5 % quantile threshold (corresponding to an rmsd-value of 0.215) for early discovery detection. This is possible as our data follows a normal distribution. Nevertheless, using a threshold means missing out on compounds with larger rmsd-values (>0.215), which could have the same inhibitory function. By taking 50 conformers into account to simulate the flexibility and to cover the conformational space, 2,500 superpositions of the two compounds were calculated and the best superposition with a minimal rmsd-value was taken for the similarity measurement. This makes the method robust with respect to conformation changes. Although the landscape of the 3D-screening shows an overall reduced similarity of inhibitors compared to the 2D-landscape, selected PARP inhibitors display high similarity when compared to the VEGFR inhibitors, confirming that it is important to take 3D in addition to 2D similarity into account to increase the hit rate. Among the inhibitors with similarity we found the anticancer agent Vatalanib, and Compound 1, a similarity not identified by different 2D fingerprint algorithms. It might be that the 2D algorithms failed to identify this similarity, because these fingerprints are based on atom labels whereas rmsd does not consider these labels. Structural comparisons as performed by the 3D algorithm used here might perform better generally, as they compensate for fragments of the molecule and their connections of different atom types. Still, there might be clusters where 2D similarity algorithms might be faster and better.

Compound 1 was identified as a direct PARP1 inhibitor in a yeast screen assay with an EC_50_ of approximately 60 μM [24]. By using the HT universal colorimetric PARP assay kit we calculated an IC_50_ value of approximately 3,000 μM. The differences in the IC_50_ values of Compound 1 can be explained by differences in the assays used as well as by differences of PARP expressed in yeast compared to an isolated PARP enzyme used in our assay. According to the colorimetric PARP assay, Vatalanib, with an IC_50_ value of 200 μM is fifteen-fold more effective than Compound 1 in inhibiting PARP. The *in silico* docking simulation indicates that Vatalanib’s additional chloride atom, which is missing in Compound 1, is in close proximity to the arginine 204 at the bottom of the binding site of PARP. A halogen bond between the nitrogen of arginine 204 might be formed, stabilising the binding position, which results in a more effective drug target inhibition. Both Vatalanib and Compound 1 have higher LibDock docking scores than 3-AB, which might be attributed to the relative small molecule size of 3-AB.

Despite better LibDock docking scores, both Vatalanib and Compound 1, are less potent than the prototype PARP inhibitor 3-AB (3-aminobenzamide), which has an IC_50_ of about 30 μM [34, 35]. Despite these comparatively high IC_50_ values of Compound 1 and Vatalanib, 10 μM of either of the drugs was able to induce γH2AX foci formation in human breast cancer cells, demonstrating DNA damage and PARP inhibition. In regard to this activity, these results again point to an *in vivo* vs. *in vitro* discrepancy with a higher bioactivity in cells compared to the enzyme assay. Due to the relative high IC_50_ of Vatalanib, Vatalanib might be of interest as a new PARP inhibitor or for drug design. In addition a putative positive side effect of Vatalanib against PARP might be important for the use of Vatalanib as a chemotherapeutical. Vatalanib doses up to 1,000 mg twice-a-day are well tolerated reaching plasma concentration in patients in the μM range. Accordingly, the 10 μM of Vatalanib, able to induce γH2AX foci, is in the rage of c_max_ plasma concentrations achieved in patients [36–39].

Vatalanib (PTK787/ZK222584) was initially described as a selective tyrosine kinase inhibitor (TKI) of VEGFR1-3. TKIs commonly have additional activity against other tyrosine kinases. Likewise Vatalanib, which at higher concentrations also inhibits other protein tyrosine kinases of the same family, such as the platelet-derived growth factor receptor beta tyrosine kinase and the c-Kit protein tyrosine kinase [19]. Interestingly, activity across other classes of drug targets have also been documented for Vatalanib. It has been shown that Vatalanib significantly inhibits aromatase and thus might cross-inhibit two important classes of targets in breast cancer [40, 41]. This “multi-targeting” activity, which might also include PARP as a target, could potentially contribute to the antitumor effect of Vatalanib and indicates that a drug’s efficacy often might not only be based on the inhibition of one target but of multiple targets [42, 43].

## Conclusion

3D similarity landscape comparison, as shown in this study, has the potential to identify new targets of known drugs. As a proof of principle, we identified Vatalanib’s additional ability to target PARP, which was demonstrated *in vitro* and *in vivo*. Thus, combined 2D and 3D similarity landscape comparison analysis can identify new functions and/or side effects of known bioactive compounds that are untraceable with 2D similarity searching alone.

## Additional files

Additional file 1: Figure S1. Complete 2D heatmap. 2D heatmap of about 10,000 inhibitors for 12 anti cancer targets. (TIFF 19184 kb)
Additional file 2: Table S1. Similar structures to PARP compounds. Details to similar structures. (DOC 170 kb)
