# Supplementary figures and images for: 2D and 3D similarity landscape analysis identifies PARP as a novel off-target for the drug Vatalanib
Source: BMC Bioinformatics. 2015 Sep 24;16:308. doi: 10.1186/s12859-015-0730-x (PMC4582733; doi:10.1186/s12859-015-0730-x)

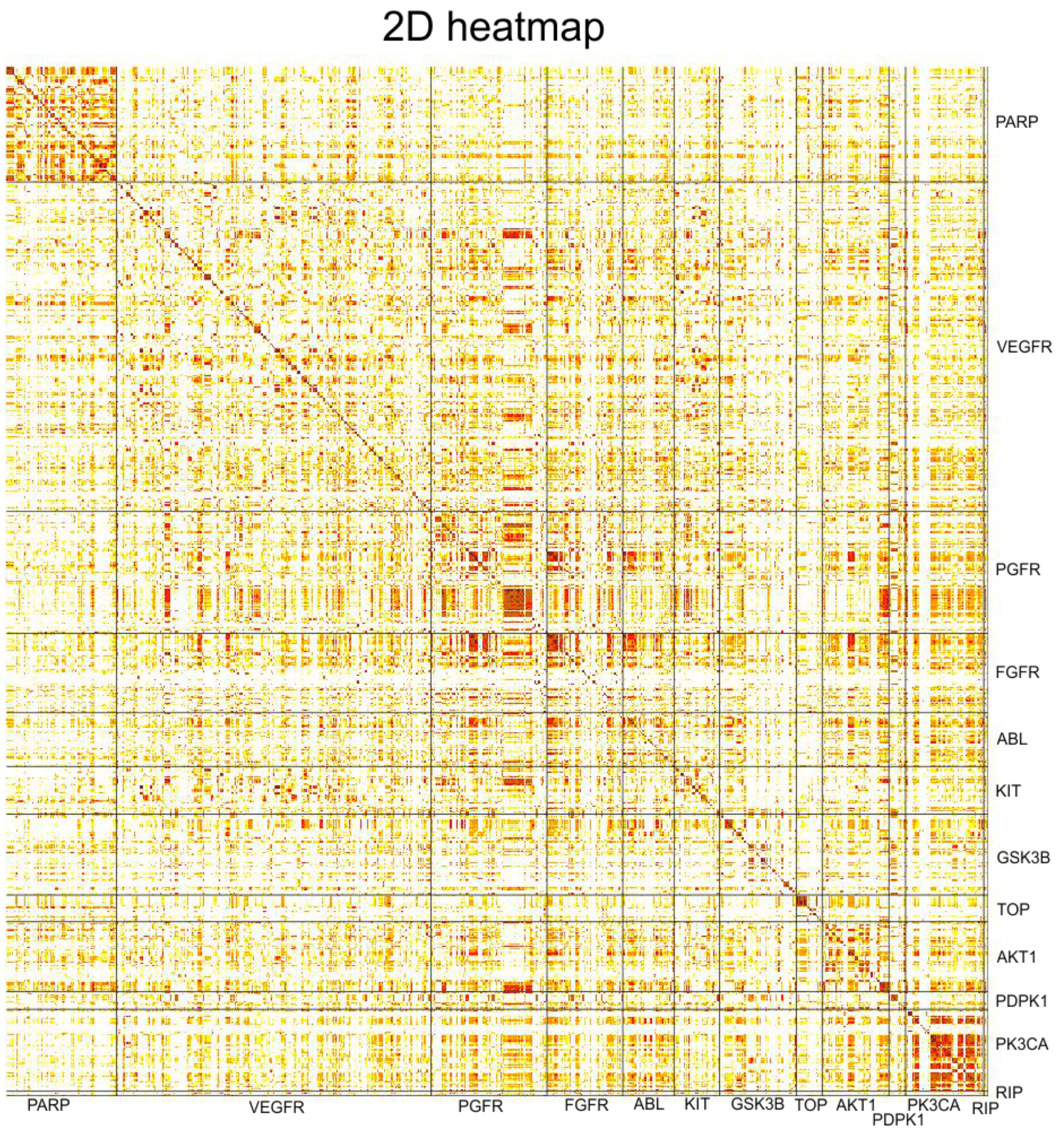

Supplement: Additional file 1: Figure S1. — Complete 2D heatmap. 2D heatmap of about 10,000 inhibitors for 12 anti cancer targets. (TIFF 19184 kb) [file 12859_2015_730_MOESM1_ESM.tif]
