# Supplementary material for: 2D and 3D similarity landscape analysis identifies PARP as a novel off-target for the drug Vatalanib
Source: BMC Bioinformatics. 2015 Sep 24;16:308. doi: 10.1186/s12859-015-0730-x (PMC4582733; doi:10.1186/s12859-015-0730-x)
Supplement: Additional file 2: Table S1. — Similar structures to PARP compounds. Details to similar structures. (DOC 170 kb) [file 12859_2015_730_MOESM2_ESM.doc]

**Table 1.** Details to similar structures

| **CID** | **Synonym** | **Structure** | **mean rmsd** | **min rmsd** |
| --- | --- | --- | --- | --- |
| 6539956 | Pyrazolo[3,4-d]pyrimidine 5 | 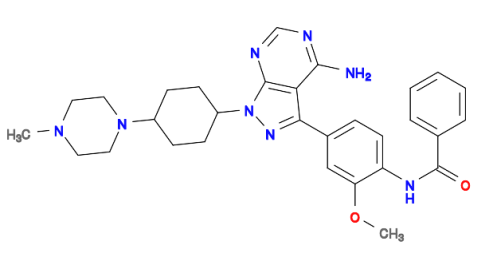 | 0,504 | 0,017 |
| 5330126 | 1,6-naphthyridine 16 | 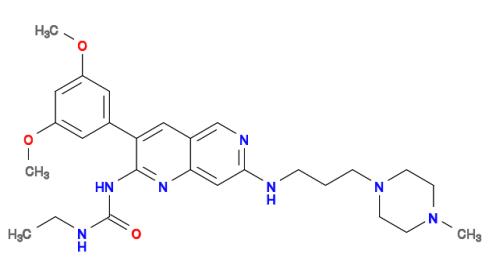 | 0,527 | 0,036 |
| 5329044 | 4-Anilinoquinazoline 18 | 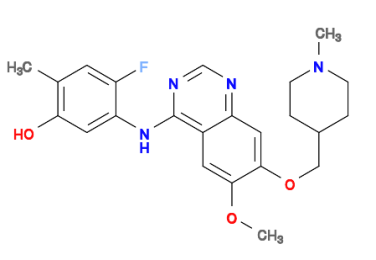 | 0,472 | 0,043 |
| 5330173 | Methoxy Aniline 5 | 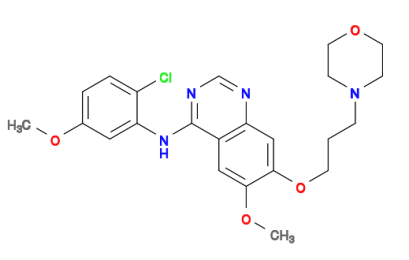 | 0,477 | 0,047 |
| 3081361 | Vandetanib | 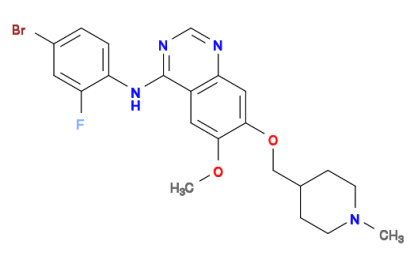 | 0,483 | 0,047 |
| 5329145 | 3-substituted indolin-2-one 11h | 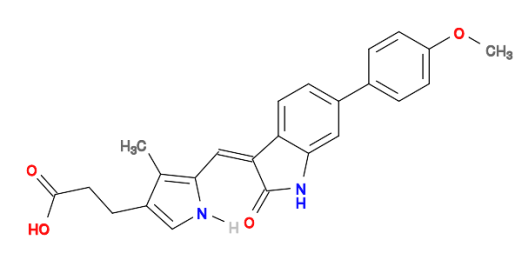 | 0,494 | 0,049 |
| 151194 | Vatalanib | 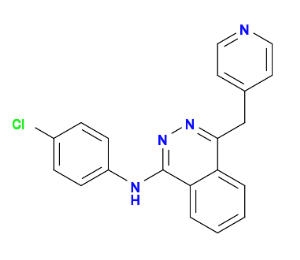 | 0,526 | 0,053 |
| 5330159 | 1,6-naphthyridine 77 | 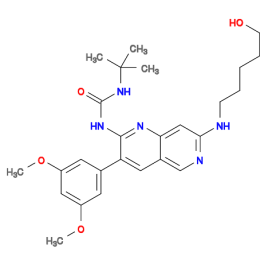 | 0,511 | 0,063 |
| 11234052 | Brivanib | 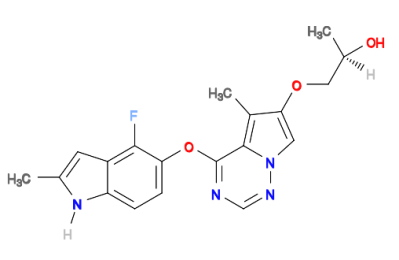 | 0,495 | 0,072 |
| 16220223 | Kinome_2108 | 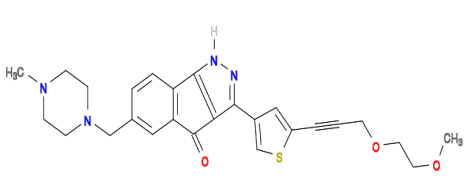 | 0,482 | 0,08 |
| 5329102 | Sunitinib | 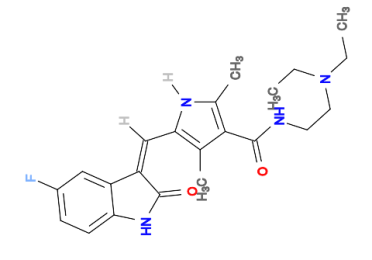 | 0,517 | 0,094 |
| 5329443 | 2,4-Disubstituted Pyrimidine 5h | 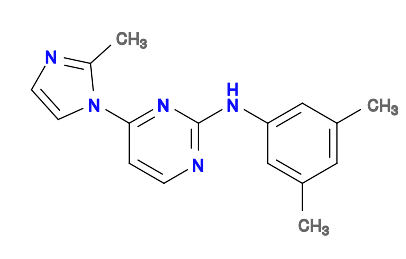 | 0,530 | 0,094 |
